# Hospital Notes—Sloughing and Destruction of the Anus and Rectum, with Prolapse of the Rectum Four Inches—Relief by Operation

**Published:** 1880-08

**Authors:** C. C. F. Gay

**Affiliations:** Surgeon to the Buffalo General Hospital


### HOSPITAL NOTES.

### SLOUGHING AND DESTRUCTION OF THE ANUS AND RECTUM, WITH PROLAPSE OF THE RECTUM FOUR INCHES.—RELIEF BY OPERATION.

SERVICE OF DR. C. C. F. GAY, SURGEON TO THE BUFFALO GENERAL HOSPITAL.

Case 1. History.—Mrs. R., aged 33 years; wife of an irregular physician; American; married four years; previous health good.

At some indefinite period since marriage her rectum began to "come down," and continued to grow worse and worse, when a strong solution of persulphate of iron (?) was applied. She was made to believe that this application caused sloughing and ablation of the rectum, and consequently, inability to control the action of her bowels. The anal outlet is so large as readily to admit the closed hand. She says she was once operated upon at Detroit, without any relief. She denies ever having specific disease, and is evidently honest and endeavors to be truthful in her statements. Preparatory to operation, a weak solution of nitrate of silver was applied, and this treatment extended over several days, and the evening previous to operation castor oil was given, and to-day, March 22, 1876, in the presence of, and assisted by the staff of the hospital, an operation was made.

The patient, etherized, was placed upon the operating table in the position for lithotomy.

The rectum, which protruded four inches, was held up in position, out of the way, by a sponge probang. Using the knife and scissors, I denuded the parts extensively, being guided by cicatricial tissue. The denuded surfaces were brought in apposition and secured by silver sutures; two of which were very deep, requiring a needle four inches in length, fixed in a strong handle, to pass them. Five more sutures were used, three of which were deep, and the remaining two superficial. A rectal tube was introduced, through which flatus might escape without imperiling union, and a flexible catheter passed into the bladder. Wide bands of adhesive plaster were applied around the body over the glutial muscles.

March 24th. On yesterday she had a small dejection from the bowels, and much retching and vomiting. Hypodermic injections morph. gr. ¼ ordered every four hours; concentrated diet to keep bowels constipated; Sigmoid catheter tried in place of elastic one, but its presence could not be tolerated, and had to be removed. Elastic tubing is attached to the catheter, to convey urine into chamber beside the bed.

April 1st. Has erysipelas extending up over the buttocks and back. To-day removed the two deep sutures; two superficial ones removed two days since. Wound suppurating. Rectal tube remains in situ.

April 4th. Removed the remaining three sutures to-day. Wound is granulating; suppurating freely. Employed larger rectal tube. She has had no evacuation of bowels since the second day; tongue dry; pulse 100; temperature 101; some abdominal tenderness. Bishop's citrate magnesia ordered and administered. She takes quinine gr. ii three times daily, McMunn's elx. opii gtt. x three times daily, brandy, wine and beef-tea. Tr. iodine painted above the erysipelatous margin. External wound not cicatrizing.

Conclude that the ailment is of syphilitic origin; accordingly ordered protiod. mercury gr. ii twice daily, and iod. pot. grs. x three times daily. Bowels have been shut up seventeen days. Open to-day.

June 3d. Wound closed, patient up, bowel does not protrude. Notwithstanding the lateness in beginning the anti-syphilitic treatment, the wound closed and the operation proved successful, but could not be expected to restore the sphinctor. A rubber pad was adjusted to the anus, and held in position by straps. The patient left the hospital well; having gained much flesh during her residence in the institution.

Remarks.—This woman was either quite ignorant of the nature of her ailment, or willing to sacrifice herself by submission to repeated operations, to conceal her husband's crime. But it is no new thing for patients to stoutly deny having had syphilis, when they ought to know, and probably do know, that the knowledge of it is indispensibly necessary to the surgeon in his proper treatment of the case. The operation came near a failure in consequence of too great reliance upon statements made both by husband and wife. Union would not have been effected but for the timely administration of mercurials and iod. pot. Undoubtedly the Detroit operation failed because of concealment of the specific origin of the rectal lesion.

